# Cell type–specific purifying selection of synonymous mitochondrial DNA variation

**DOI:** 10.1073/pnas.2505704122

**Published:** 2025-07-24

**Authors:** Caleb A. Lareau, Patrick Maschmeyer, Yajie Yin, Jacob C. Gutierrez, Ryan S. Dhindsa, Anne-Sophie Gribling-Burrer, Sebastian Zielinski, Yu-Hsin Hsieh, Lena Nitsch, Veronika Dimitrova, Benan Nalbant, Frank A. Buquicchio, Tsion Abay, Robert R. Stickels, Jacob C. Ulirsch, Patrick Yan, Fangyi Wang, Zhuang Miao, Katalin Sandor, Bence Daniel, Vincent Liu, Paul L. Mendez, Petra Knaus, Manpreet Meyer, William J. Greenleaf, Anshul Kundaje, Redmond P. Smyth, Mathias Munschauer, Leif S. Ludwig, Ansuman T. Satpathy

**Affiliations:** ^a^Computational and Systems Biology Program, Memorial Sloan Kettering Cancer Center, New York, NY 10065; ^b^Department of Pathology, Stanford University, Stanford, CA 94305; ^c^Berlin Institute of Health at Charité—Universitätsmedizin Berlin, Berlin 10117, Germany; ^d^Max-Delbrück-Center for Molecular Medicine in the Helmholtz Association, Berlin Institute for Medical Systems Biology, Berlin 10115, Germany; ^e^Department of Pathology and Immunology, Baylor College of Medicine, Houston, TX 77030; ^f^Jan and Dan Duncan Neurological Research Institute, Texas Children’s Hospital, Houston, TX 77030; ^g^Department of Molecular and Human Genetics, Baylor College of Medicine, Houston, TX 77030; ^h^Helmholtz Institute for RNA-based Infection Research, Helmholtz-Center for Infection Research, Würzburg 97080, Germany; ^i^Department of Biology, Chemistry, Pharmacy, Freie Universität Berlin, Berlin 14195, Germany; ^j^Biological and Biomedical Sciences Program, Harvard Medical School, Boston, MA 02115; ^k^Illumina AI Laboratory, San Diego, CA 92122; ^l^Department of Genetics, Stanford University, Stanford, CA 94305; ^m^Boston Children’s Hospital and Harvard Medical School, Boston, MA 02115; ^n^Department of Computer Science, Stanford University, Stanford, CA 94305; ^o^Department of Infectious Diseases, Center for Integrative Infectious Disease Research, Heidelberg 69120, Germany; ^p^Department of Medicine, Heidelberg University, Heidelberg 69120, Germany; ^q^Parker Institute for Cancer Immunotherapy, San Francisco, CA 94129

**Keywords:** single-cell, mitochondria, selection, immunology

## Abstract

While somatic variants are well-characterized drivers of tumor evolution, their influence on cellular fitness in nonmalignant contexts remains understudied. We identified a mosaic synonymous variant (m.7076A > G) in the mitochondrial DNA (mtDNA)-encoded cytochrome c-oxidase subunit 1 (MT-CO1, p.Gly391=), present at homoplasmy in 47% of immune cells from a healthy donor. Single-cell multiomics revealed strong, lineage-specific selection against the m.7076G allele in CD8^+^ effector memory T cells, but not other T cell subsets, mirroring patterns of purifying selection of pathogenic mtDNA alleles. The limited anticodon diversity of mitochondrial tRNAs forces m.7076G translation to rely on wobble pairing, unlike the Watson–Crick–Franklin pairing used for m.7076A. Mitochondrial ribosome profiling confirmed stalled translation of the m.7076G allele. Functional analyses demonstrated that the elevated translational and metabolic demands of short-lived effector T cells (SLECs) amplify dependence on MT-CO1, driving this selective pressure. These findings suggest that synonymous variants can alter codon syntax, impacting mitochondrial physiology in a cell type–specific manner.

## Significance

We identify the cell type–specific selection of T cells with a synonymous mitochondrial DNA variant in *MT-CO1,* reminiscent of the purifying selection of pathogenic alleles. Mechanistically, we implicate the limited mitochondrial tRNA pool to stall effective ribosomal translation of “wobble” codons. Together, this suggests a mechanism of functional mtDNA variation that is enabled by the distinct tRNA pool. The results from a specific variant inferred from single-cell analyses provides context for patterns of mutation observed in the germline.

The human body is composed of thousands of distinct cell types with highly specialized functions. Notably, the impact of pathogenic genetic variants on functional phenotypes may be pronounced in specific cell states but silent in others, indicative of demands of otherwise ubiquitous molecular processes, including translation and oxidative phosphorylation (1, 2). Recent single-cell sequencing efforts have charted the effects of state-specific variant functions across human cell types, predominantly through studying common variant effects via single-cell expression quantitative trait loci mapping (3, 4). Other approaches use single-cell genotyping to discern transcriptional and epigenetic impact of somatic variants across different lineages in the hematopoietic system (5, 6). While these and other studies enable a new annotation of functional variation in the human genome, our understanding of cell type–specific functional DNA variation remains underexplored.

Recently, we have introduced single-cell multiomic methods that detect genetic variants in mitochondrial DNA (mtDNA) with concomitant measures of cell state, including accessible chromatin (7), gene expression (8), and/or protein abundances (9, 10). While these methods have broadly enabled lineage tracing across human tissues, their application to congenital mitochondrial disorders has revealed previously unappreciated state-specific impacts of pathogenic single nucleotide variation (11, 12) and single large-scale mtDNA deletions (SLSMD) across human immune cells (13, 14). Noting mtDNA is present at approximately 100 copies per cell in lymphocytes, we and others have shown that distinct immune cell lineages differentially tolerate loss-of-function (LOF) mutations, including the notable purifying selection of the m.3243A > G mutation in T cells (11, 15). More recently, we have refined these observations and demonstrated CD8^+^ effector-memory T cells (CD8^+^ TEM) to be particularly vulnerable to LOF heteroplasmy (13), suggesting that activation and expansion of naive CD8^+^ T cells, in particular, require intact mitochondrial genetic integrity, relative to other immune cells as further corroborated by other groups including in a mouse model of mitochondrial disease (16, 17).

Here, we report the identification of a mosaic synonymous mtDNA mutation, m.7076A > G (*MT-CO1*:p:Gly391=), in peripheral blood mononuclear cells (PBMCs) from a healthy donor. While we observe similar allelic heteroplasmy of this variant in all hematopoietic lineages, we observed a depletion of the m.7076G allele specifically in the effector memory CD8^+^ T cell compartment, reminiscent of the selection of diverse pathogenic mtDNA variants (11, 13). We demonstrate that due to the limited diversity of the transfer RNA (tRNA) pool in mitochondria, this synonymous mutation requires translation via the super-wobble effect, where a 5’ uracil in the anticodon can decode all four nucleotides in the 3’ codon. While capable of translation, the U-G wobble base pairing stalls mitochondrial ribosomes, thereby impeding CD8^+^ T cell differentiation. Our study provides evidence of somatic mtDNA mosaicism impacting cell type–specific metabolic requirements, motivating continued study of somatic evolution via scalable single-cell technologies.

## Results

### A Mosaic Synonymous mtDNA Variant that Is Selected Against in the CD8^+^ T Cell Compartment.

**Fig. 1.**
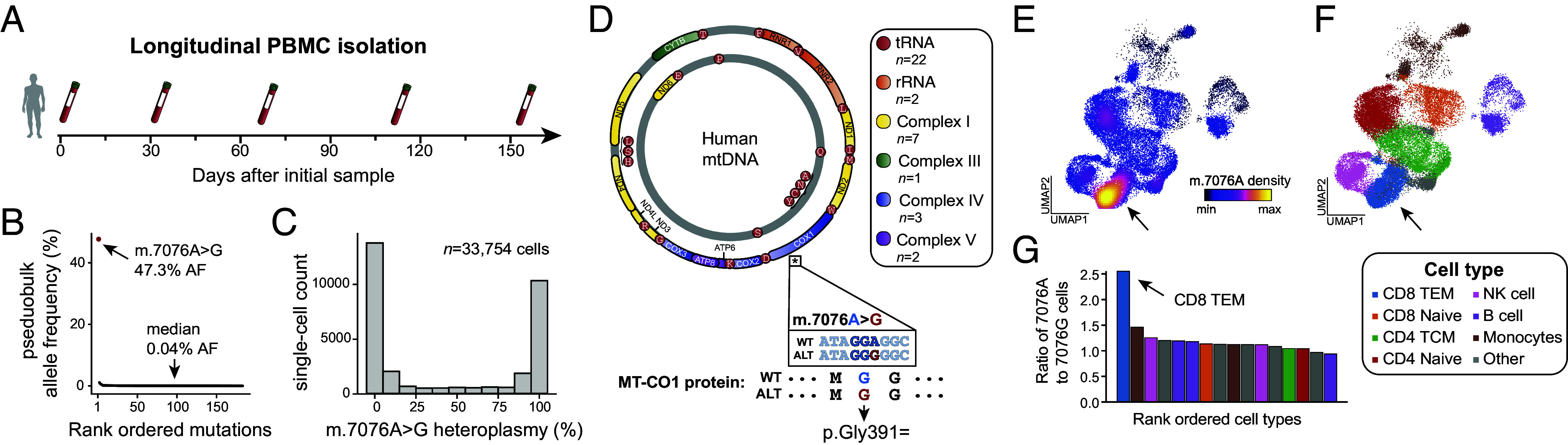
Identification of a mosaic synonymous mtDNA variant with a CD8^+^ T cell restricted selection bias. (*A*) Schematic of longitudinal peripheral blood samples obtained from a healthy donor. A total of 5 draws spanning 150 d were taken and processed with the mtscATAC-seq assay. (*B*) Summary of somatic mtDNA mutations called from aggregated draws. The overall median heteroplasmy is noted, as well as the m.7076A > G allele that is present at a 47.3% pseudobulk heteroplasmy. (*C*) Distribution of single-cell heteroplasmy across all cells profiled for the m.7076A > G allele. (*D*) Schematic of the mitochondrial genome with genes contributing to indicated complexes of the respiratory chain (I, III, IV, and V) being color-coded. The asterisk under the *MT-CO1* genes denotes the position of the m.7076 allele. Annotation of the mutation, including the protein consequence (p.Gly391=) of the synonymous variant is shown below. (*E*) Uniform manifold approximation and projection (UMAP) of accessible chromatin profiles of PBMCs assayed via mtscATAC-seq colored by the density of the m.7076A (wildtype) allele. (*F*) Cluster annotation and cell type labeling of the same cells as in (*E*). The arrow indicates the CD8^+^ T effector memory (CD8^+^ TEM) population. (*G*) Ratio of homoplasmic cells with wildtype m.7076A to mutant m.7076G variants across indicated cell state clusters. The arrow highlights the CD8^+^ TEM cell state as the population with the greatest skew (*P* < 2.2e−16; binomial test).

We sampled PBMCs over a five-month longitudinal course of a healthy donor with the mitochondrial scATAC-seq assay (mtscATAC-seq) to profile cell state and mtDNA genotyping information from the same single cells, yielding 33,754 high-quality single cells (Fig. 1*A*). Analysis of the homoplasmic germline variants revealed this individual to be part of haplogroup M38a (which includes m.7076A), consistent with self-reported ancestry of the individual. Concordant with our prior observations from native hematopoiesis in healthy individuals (7), we identified 183 somatic mtDNA variants with a median allele frequency of 0.04% in pseudobulk (Fig. 1*B*; *Materials and Methods*). Notably, we observed that one highly heteroplasmic variant, m.7076A > G, was present at 47.3% allele frequency, potentially reflecting germline mosaicism or of early developmental origin (18). The m.7076A > G variant is a synonymous variant (p.Gly391=) in the mitochondrial cytochrome c oxidase subunit 1 (*MT-CO1*), a gene required for complex IV activity during oxidative phosphorylation (Fig. 1 *C* and *D*). The majority of the 33,754 profiled cells had either 0% or 100% heteroplasmy (Fig. 1*C* and *SI Appendix*, Fig. S1*A*), a pattern that we previously observed for a noncoding mutation m.16260C > T in an independent healthy donor (9) and others have identified in other somatic cells (19). While m.7076A > G is present as a homoplasmic variant in 0.2 to 0.9% of the population (20, 21), the variant does not define a specific mitochondrial haplogroup. Still, approximately 1 in 15,000 individuals carry a highly heteroplasmic m.7076A > G allele (10 to 90% allele frequency) similar to this donor (20, 21) (*Materials and Methods*).

Leveraging the chromatin accessibility modality of the mtscATAC-seq dataset, we used a dictionary learning strategy to infer the cell states for each cell that was concomitantly genotyped (*Materials and Methods*). Aggregating over all timepoints profiled, we observed a marked depletion of cells homoplasmic for the mutant m.7076G allele in a subset of CD8^+^ TEM, a depletion specific to this cell state (Fig. 1 *E* and *F* and *SI Appendix*, Fig. S1 *B*–*D*). In other words, CD8^+^ TEMs had a marked increase of cells with the wildtype m.7076A allele, suggestive of lineage-specific selection pressure against cells with the mutant m.7076G allele (Fig. 1*G*). Notably, these findings mirror our recent observations of purifying selection against pathogenic mtDNA mutations in CD8^+^ TEM cells in individuals with congenital mitochondriopathies, including patients with Mitochondrial Encephalopathy, Lactic Acidosis, and Stroke-like episode (MELAS) (11, 12) and Pearson Syndrome (13, 14), which is driven by the distinct demand for OXPHOS capacity during T cell proliferation and differentiation after activation (22, 23), refining decades of observation of purifying selection of pathogenic alleles in peripheral blood (15).

### Altered T Cell State-Specific Gene Expression due to m.7076A > G.

**Fig. 2.**
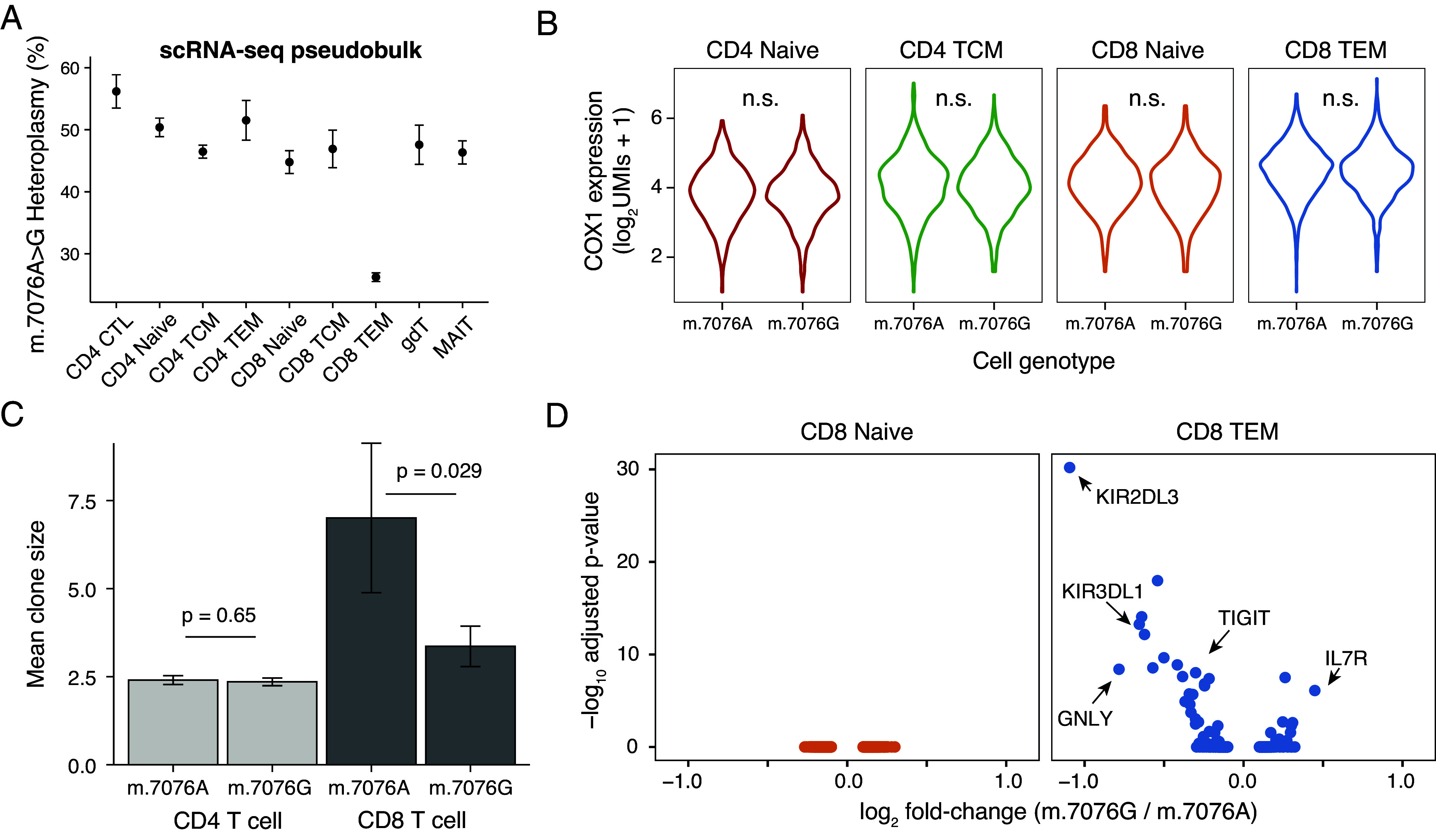
Stable expression of *MT-CO1* transcript but altered clone size of CD8^+^ effector memory T cells carrying the mutant m.7076G allele. (*A*) Heteroplasmy of m.7076A > G in indicated T cell subpopulations based on scRNA-seq. The size of each dot is scaled by the abundance of cells in each cell state. (*B*) Comparison of *MT-CO1* expression across indicated cell states stratified by the m.7076A and G alleles. *P*-values from a Wilcox test comparing log_2_ *MT-CO1* UMI counts per cell were not significant at type I error of 0.05. (*C*) Comparison of TCR clone sizes (number of cells per clone) between CD4^+^ and CD8^+^ T cells with homoplasmic m.7076A or m.7076G alleles. *P*-values are shown for a Wilcox test comparing clone sizes which were significant at type I error of 0.05. (*D*) Differential gene expression of all genes between cells with homoplasmic wildtype m.7076A vs. mutant m.7076G within the indicated CD8^+^ T cell compartments. 0 genes were differentially expressed in naive CD8^+^ T cells whereas 32 were differentially expressed in CD8^+^ TEM cells, including the 5 highlighted in the text. No other differentially expressed genes were observed in other T cell subsets. Multiple hypothesis testing: per subtype Bonferroni-adjusted *P*-value.

As synonymous variants alter the sequence of DNA but retain the amino acid sequence of the encoded protein, these alleles do not tend to have a functional impact and are typically annotated as “silent” mutations. However, differential effects of distinct tRNAs decoding synonymous codons and alterations in enzyme structure and function due to synonymous mutations have been reported (24), including in human tumor progression (25, 26). Thus, we hypothesized that the synonymous variant m.7076A > G impacts cellular fitness following T cell activation/differentiation, which would be analogous to our characterization of selection in the T cell compartment for other variants (11, 13). To examine the possibility of this mutation impacting *MT-CO1* mRNA expression, we performed single-cell RNA-seq (scRNA-seq) profiling of 17,337 T cells derived from PBMCs derived from this donor. We verified the specific depletion of the m.7076G allele in the CD8^+^ TEM compartment, but not other T cell subsets (Fig. 2*A*). As a recent study attributed functional synonymous variation in yeast to differences in gene expression (27), we compared the abundance of the *MT-CO1* transcript between cells with homoplasmy for either the wildtype or mutant allele (*Materials and Methods*). Within any of these cell subsets, we did not observe significant differences in expression, suggesting that the synonymous variant does not impact *MT-CO1* stability or expression (Fig. 2*B*).

Using concomitant single-cell T cell receptor (TCR) sequencing, we examined clone sizes of CD4^+^ and CD8^+^ T cells with either m.7076 allele. As expected, TCR clones were effectively mutually exclusive for either m.7076 allele, confirming the prevalence of the mutant allele prior to TCR diversification (*SI Appendix*, Fig. S2*A*). We observed a diminished clone size in the CD8^+^ but not CD4^+^ T cell compartment in cells with the mutant m.7076G allele (Fig. 2*C*), suggesting reduced fitness or proliferative capacity of mutant CD8^+^ T cells to clonally expand. Further, we performed differential gene expression analyses across T cell subsets between wildtype and mutant m.7076 cells. Whereas all CD4^+^ subsets and CD8^+^ naive subsets had no differentially expressed genes between cells with distinct genotypes, we observed 32 differentially expressed genes between wildtype and mutant CD8^+^ TEM cells (Fig. 2*D*). While cytotoxic genes including *GNLY*, *KIR2DL3*, and *KIR3DL1* were down-regulated, *IL7R*, a marker of naive T cells, was more highly expressed in cells with the mutant m.7076G allele. Additional unsupervised analyses revealed distinct cell states within the CD8^+^ TEM compartment, including highly clonal wildtype m.7076 allele populations expressing specific TCRs (*SI Appendix*, Fig. S2 *B* and *C*). However, these highly expanded clones can not alone explain the observed depletion of the mutant allele in CD8^+^ TEM (*SI Appendix*, Fig. S2*D*; *Materials and Methods*). Though the mutant m.7076A > G allele does not preclude the possibility of CD8^+^ TEM differentiation, we conclude that mutant cells are at a competitive disadvantage to expand and acquire fully differentiated cytotoxic T cell–like phenotypes as evidenced by gene expression defects.

**Fig. 3.**
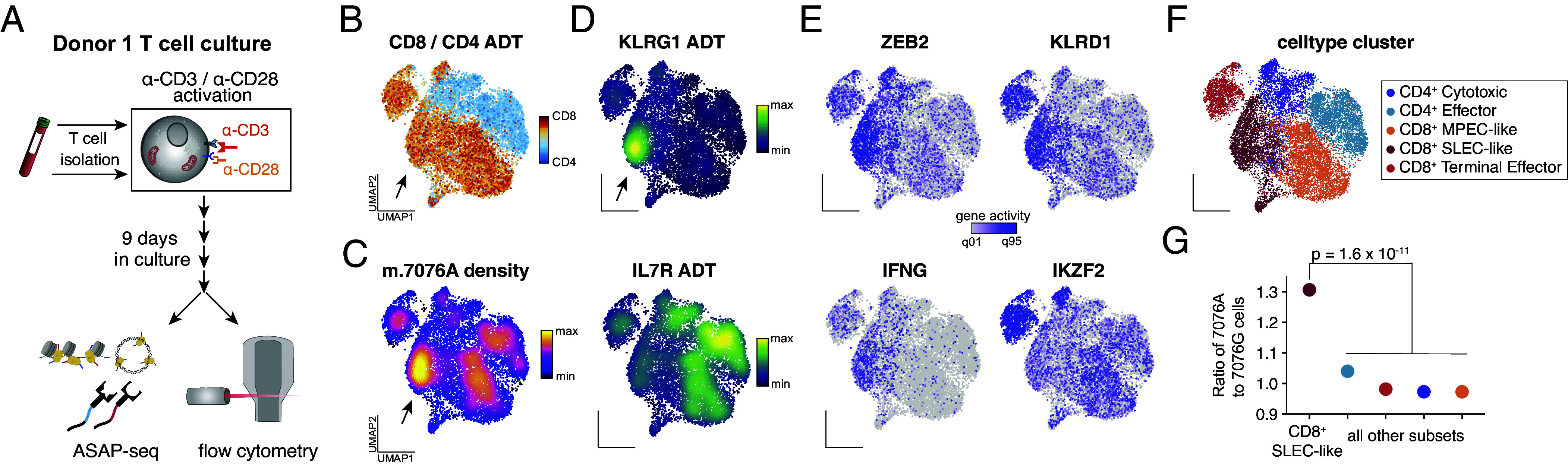
In vitro activation of T cells refines cell states depleted of the mutant m.7076G allele. (*A*) Schematic of experimental design. T cells were isolated from Donor 1, in vitro activated, and cultured for 9 d before profiling via flow cytometry and ASAP-seq. (*B*) UMAP of accessible chromatin profiles and projected ratio of CD8 over CD4 antibody-derived tags from day 9 cells profiled via ASAP-seq. The arrow indicates a population highly enriched for the m.7076A (wildtype) allele. (*C*) Same as (*B*) but colored by the density of the m.7076A (wildtype) allele. (*D*) UMAP embedding colored by KLRG1 (*Top*) and IL7R (*Bottom*) antibody tag density. (*E*) UMAP colored by selected gene activity scores for four indicated gene loci. (*F*) UMAP colored by indicated cell state cluster. (*G*) Ratio of wildtype m.7076A to mutant m.7076G cells within indicated cell states. *P*-value represents the statistical significance of a two-sided binomial test statistic.

To further investigate the CD8^+^ T cell deficiencies incurred by this variant, we stimulated PBMC-derived T cells and examined cell phenotypes using flow cytometry and ATAC with Selected Antigen Profiling via sequencing (ASAP-seq) that extends the mtscATAC-seq to coquantify surface marker profiles via oligo-barcoded antibodies (Fig. 3*A*). Leveraging the multimodal measurements, we again observed a concentration of the wildtype m.7076A allele in a specific CD8^+^ T cell cluster (Fig. 3 *B* and *C*). Interestingly, this cluster is characterized by high KLRG1 surface protein expression but depleted of IL7R protein, consistent with the transcriptomics data (Fig. 3 *D* and *E*). Gene scores showed highly accessible chromatin in the *ZEB2*, *IFN-γ*, and *KLRD1* loci, suggestive of a CD8^+^ short-lived effector cell (SLEC)-like population that is 1.3× enriched for cells with the wildtype m.7076A allele compared to other T cell subsets (Fig. 3 *F* and *G*; *P* = 1.6 × 10^−11^; binomial test). Thus, our T cell culture experiments confirm mutant m.7076A > G cells to be impaired in their ability to attain specific CD8^+^ T cell states in vitro.

### Short-Lived Effector Cells Show Distinct Metabolic Capacities.

**Fig. 4.**
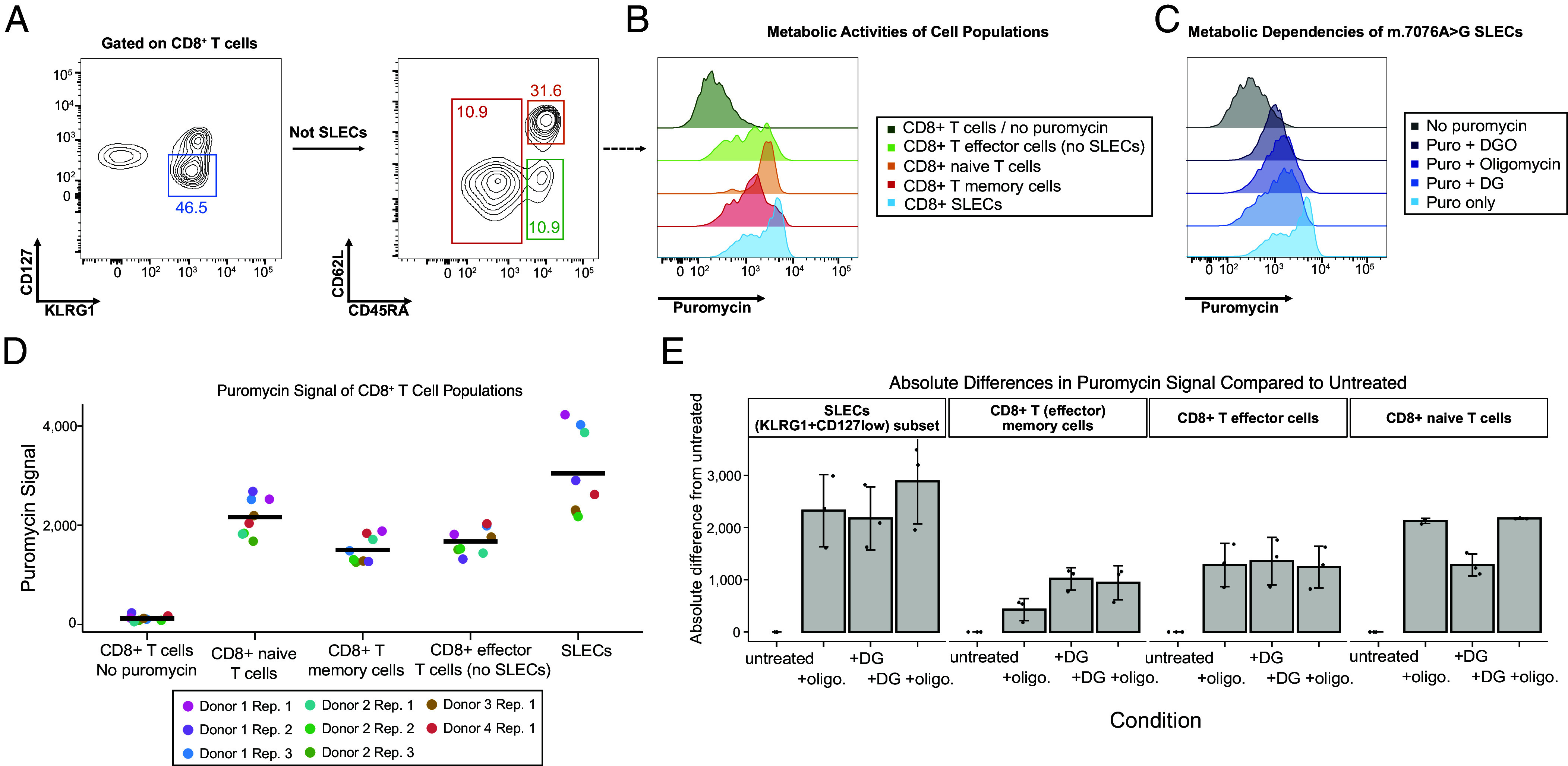
SLECs exhibit high metabolic activity and dependence on OXPHOS. (*A*) PBMCs from four healthy donors, including Donor 1 were analyzed using SCENITH. Gating strategy to distinguish CD8^+^CD45RA^+^CD62L^+^ naive, CD8^+^CD45RA^+^CD62L^-^ effector (SLECs not included), CD8^+^CD45RA^-^ memory (primarily CD62L^–^ effector/memory), and CD8^+^KLRG1^+^CD127^low^ T cells (SLECs) is shown. (*B*) Representative histograms showing puromycin levels indicative of cell type–specific translational/metabolic activities. (*C*) SLEC dependencies after glycolysis (DG), OXPHOS (Oligomycin) or both (DGO) are shown. Cells not treated with puromycin (no puromycin) were used as negative control. (*D*) Quantification of puromycin levels across CD8^+^ T cell subsets. Bulk CD8^+^ T cells without puromycin treatment served as a negative control. (*E*) T cells from the m.7076A > G donor were treated with OXPHOS (oligo), glycolysis (DG), or both inhibitors (DGO) to quantify absolute differences in puromycin incorporation to probe relative metabolic dependencies. Bar graphs display the reduction in puromycin incorporation of CD8^+^ T cell subsets in comparison to samples without inhibitor treatment. Data of 3 technical replicates are shown.

To corroborate the cell-state-specific nature of the selection, we leveraged in vitro puromycin incorporation assays to probe translational activity and flow cytometric quantification to probe possible distinct metabolic cellular requirements of CD8^+^ T cell states via SCENITH as previously described (28). Notably, SLECs showed the highest levels of absolute puromycin incorporation after 30 min relative to other T cells, indicating a higher metabolic activity (Fig. 4 *A*–*D*). SLECs also showed the highest absolute difference upon brief (30 min.) nontoxic metabolic inhibitor treatments (*SI Appendix*, Fig. S3 *A* and *B*), including 2-deoxy-glucose (DG) and oligomycin (O), potent inhibitors of glycolysis and oxidative phosphorylation, respectively. Specifically, SLEC translation was equally impaired following DG, and O-treatment, which was further exacerbated when combined (DGO), indicating a strong dependency of SLECs on mitochondrial function and aerobic glycolysis (Fig. 4*E*). As such, the selection of SLECs with the mutant m.7076G allele is likely attributable to a higher metabolic activity relative to other CD8^+^ T cell states, thereby driving the observed selection phenotypes.

### Examination of Nuclear Mosaic Variation.

To further exclude potential confounding elements, we sought to examine whether a separate nuclear variant cosegregated with the mtDNA variant and could alternatively explain the apparent selection phenotype. Using our refined T cell surface marker phenotypes, we sorted four populations from PBMCs of the donor and performed deep whole-exome sequencing on these populations (420 to 460× coverage; *SI Appendix*, Fig. S3 *C* and *D*; *Materials and Methods*). We included mitochondrial-specific probes as part of the exome panel and were able to genotype m.7076A > G in all libraries, with heteroplasmy ranging from 11 to 52% as expected from our mtscATAC-seq analyses (*SI Appendix*, Fig. S3*E*). Using these ultradeep exome libraries, we devised an analytical strategy to determine whether specific nuclear mutations were cosegregating with either the m.7076A or m.7076G allele but could identify no variants of predicted impact (*SI Appendix*, Fig. S3 *F* and *G*; *Materials and Methods*). These analyses support that the selective phenotype observed in PBMCs is attributable to a putative functional effect of m.7076A > G.

### Altered Wobble-Dependent Translation of m.7076A > G.

**Fig. 5.**
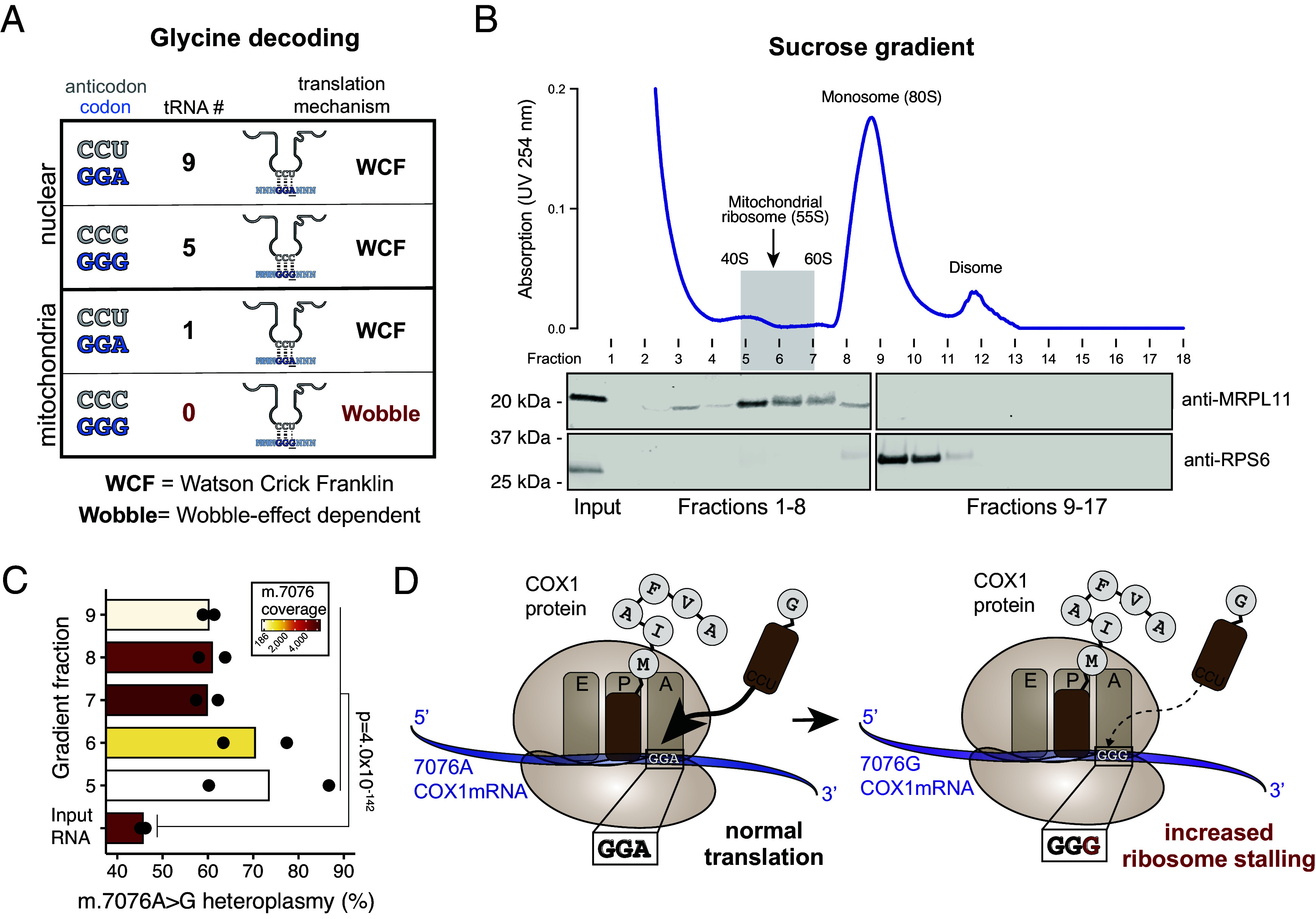
Mitochondrial ribosome profiling reveals translational stalling of the mutant m.7076A > G allele. (*A*) Schematic of codon, anticodon, tRNA, and codon:anticodon recognition mechanisms for glycine in the human nuclear and mitochondrial genomes. (*B*) Polysome profile following sucrose gradient and western blots of isolated fractions for mitochondrial ribosome profiling. MRLP11 and RPS6 were blotted to identify enrichment of mitochondrial and cytoplasmic ribosomes, respectively. (*C*) Summary of heteroplasmy from ribosome profiling libraries [fractions 5 to 9; see panel (*B*)] showing a relative increase of the mutant m.7076A > G allele in ribosomal bound fractions versus input RNA. Statistical significance was determined using a Fisher’s exact test of 7076A and G read counts summed between replicates. (*D*) Schematic of the functional effect of the synonymous m.7076A > G variant. Due to decreased codon:anticodon affinity of the m.7076A > G allele, there is an increase in stalling of the *MT-CO1* transcript, prohibiting effective translation.

Given no alterations in *MT-CO1* transcript abundance (Fig. 2), we hypothesized a posttranscriptional mechanism to be responsible for the mutant functional effects. As synonymous codons may be decoded by independent tRNAs, we revisited the distinct pool of tRNAs in mitochondrial translation compared to nuclear-encoded tRNAs for cytoplasmic protein biosynthesis. Specifically, the nuclear genome redundantly encodes multiple tRNA loci for all possible codons, but only 22 tRNAs are ordinarily transcribed in the human mitochondrial genome. Thus, only a single glycine-specific tRNA (tRNA^Gly^) is encoded in the mitochondrial genome to decode p.Gly391 via the wildtype GGA codon (m.7076A) or the mutant GGG codon (m.7076A > G; Fig. 5*A*). As such, decoding via the single mt-tRNA^Gly^ of the wildtype GGA allele occurs via canonical Watson–Crick–Franklin (WCF) base-pairing but requires wobble-dependent translation of the mutant GGG codon (Fig. 5*A*). Although the GGG codon is relatively depleted in the mitochondrial genome compared to the nuclear genome (*SI Appendix*, Fig. S4 *A* and *B*), this codon occurs at 34 positions within the 13 peptide-encoding open reading frames, suggesting the mitochondrial translational machinery to be generally capable of decoding this codon. Based on our cell state mapping experiments, we hypothesized that the substantial requirement for oxidative phosphorylation during CD8^+^ T cell activation, differentiation, and proliferation may present a distinct metabolically vulnerable state toward CD8^+^ TEM cell state transition that is sensitive to even subtle changes in oxidative phosphorylation capacity.

To examine this possibility, we stimulated PBMCs in the presence of αCD3/αCD28 beads and IL-2 and assessed mitochondrial translational efficiency (*Materials and Methods*). We isolated fractions along a sucrose gradient. To verify fractions with mitochondrial ribosomes, we performed western blot analysis detecting the mitochondrial ribosomal protein L11 (MRPL11) protein and prepared libraries of input RNA and sucrose gradient fractions 5 to 9, for mitochondrial ribosome profiling by sequencing (MitoRiboSeq; Fig. 5*B*; *Materials and Methods*) (29). Hypothesizing the altered codon syntax of the mutant m.7076A > G allele to impair translational decoding, the mutant G allele would be relatively enriched in the ribosome-protected fragments (RPFs) compared to the total mRNA input. Indeed, across all 10 fractions (5 per biological replicate), we computed a translation pause ratio, defined as the fraction of reads from ribosome profiling over the RNA-seq libraries, and observed that the mutant m.7076A > G allele was enriched over the input material (Fig. 5*C*, *SI Appendix*, Fig. S4*C*, mean 33% increase; *P* = 4.0 × 10^−142^), which was further replicated in an independent experiment (Fig. S4*D*, 3 additional replicates; mean 37% increase; *P* = 3.1 × 10^−24^). Specifically, the wildtype and mutant alleles were present at similar transcript levels (54.3% m.7076A vs. 45.7% m.7076G; ratio 1.19) compared to the mtscATAC-seq based genotyping results (47.3% m.7076G) further confirming *MT-CO1* mRNAs abundance to be relatively unaltered. However, among ribosomal protected fragments, the mutant allele was substantially more abundant (39.1% wildtype m.7076A vs. 60.9% mutant m.7076A > G; ratio 0.64), strongly evident of translational stalling around the mutant codon (*SI Appendix*, Fig. S4*C*). Thus, the limited diversity of the tRNA pool prohibits efficient translation of the mutant m.7076A > G allele, otherwise requiring the super-wobble effect (where a 5’ uracil in the anticodon can decode all four nucleotides in the 3’ codon) to effectively decode the mutant GGG. As a consequence, translation of *MT-CO1* is stalled via a mechanism consistent with reports of super-wobble model systems (Fig. 5*D*) (30), thereby creating a functional phenotype impacting CD8^+^ T cell differentiation.

### Germline Mutations Passively Optimize Codon Affinity.

Given the overall restricted mitochondrial tRNA pool, we evaluated the potential impact of synonymous mtDNA variation beyond the mutant m.7076A > G allele, noting the limited tRNA pool decoding all amino acids in the mitochondria compared to their nuclear counterparts (*SI Appendix*, Fig. S5 *A* and *B*). We considered all possible synonymous mutations across the mitochondrial genome, totaling 8,284 variants across 13 polypeptide-encoding genes (*SI Appendix*, Fig. S5*C*). In our ontogeny, we annotated variants based on whether the reference and alternate alleles use canonical WCF or wobble base-pairing, leading to four possible classifications for synonymous variants: WCF → WCF, WCF → Wobble, Wobble → WCF and Wobble → Wobble (*SI Appendix*, Fig. S5*C*; *Materials and Methods*). From our annotation, we observed 48% of codons in the mitochondrial genome require wobble-dependent translation based on the mtDNA reference, suggesting that wobble-dependent translation could impact additional sites beyond m.7076, potentially in a similar cell-type-specific function (*Materials and Methods*).

To evaluate the potential impact of this annotation on mtDNA variation interpretation, we identified 87 common synonymous variants linked to the phylogeny of human mitochondrial haplogroups and examined their proportions in light of our ontogeny (*Materials and Methods)*. Here, we observed a >2.5 fold increase of Wobble → WCF variants (observed: 56.3%; null: 22.0%; *P* = 2.8 × 10^−14^), suggesting that variation linked to human mtDNA haplotypes for increased WCF pairing compared to the most recent common human ancestor is an ongoing evolutionary process (*SI Appendix*, Fig. S5*D*). Next, we considered interspecies annotations of conservation, requiring a per-position analysis rather than a per-variant analysis (*Materials and Methods*). We classified each of the wobble-position nucleotides in the mitochondrial genome into three categories: positions that could variably lead to missense or synonymous variants (*n* = 2,158), WCF reference alleles that become wobble in all scenarios (*n* = 790), or wobble reference alleles that, when mutated, are either wobble or WCF (*n* = 1,250). Using both a 100-species phyloP score (31), we next assessed the evolutionary conservation of each codon class. Notably, we observed a significant decrease in phyloP score specifically for positions that require the wobble effect for translation in the reference genome, suggesting that these loci are under accelerated evolution compared to already optimal WCF pairings (*SI Appendix*, Fig. S5*E*; *P* = 1.03 × 10^−111^; Wilcoxon test).

Together with our characterization of the m.7076 allele, these results suggest that the mitochondrial genome may experience selective pressure for codon optimality. To further evaluate this annotation, we stratified our annotation by the type of nucleic acid change per variant, noting that transitions (A > G, G > A, C > T, and T > C) are far more commonly observed somatic mutations in the mtDNA genome across tissues due to pervasive deamination (32). Here, we observed that G > A and T > C mutations, the two most common types of nucleotide changes in the mitochondrial genome, often result in Wobble → WCF changes that yield more optimized codon:anticodon pairings (*SI Appendix*, Fig. S5 *F* and *G*). This annotation is consistent with previous analyses that linked mutational signatures and codon imbalances (32). Thus, while we note an enrichment of observed mutations that optimize codon affinity, this codon syntax is likely a passive consequence of the tRNA decoding logic and nucleotide changes associated with deamination. Taken together, these results reflect that codon syntax influences mitochondrial function across somatic and evolutionary time scales.

## Discussion

Mapping somatic DNA mutations across healthy tissues revealed hundreds of somatic mutations that undergo positive selection in variable physiologic contexts (33–35). However, these mutations are infrequently shared between multiple tissues, suggesting that many somatic variants contribute to cell state heterogeneity in a context-specific manner (36). Here, we characterized a heteroplasmic somatic mtDNA variant m.7076A > G in the peripheral blood of a healthy individual to discern a functional role for a synonymous mutation. Consistent with our prior work of purifying selection against pathogenic mtDNA variants (11, 13), cells harboring the m.7076G allele appear functionally distinct from their m.7076A counterparts, which alters their clonal expansion, cell state, and in vivo abundance specifically in the CD8^+^ T cell compartment. Ultimately, our characterization of the wobble-dependent functional effect of this allele enables a previously underappreciated annotation of the mitochondrial genome. We suggest that some, but not all, other possible synonymous mutations may similarly impact translational efficiency and buffer cell state phenotypes and/or transitions with variable oxidative phosphorylation demands. While naturally occurring mutations analogous to m.7076A > G appear rare and will require larger-scale population efforts to facilitate their identification, our scalable single-cell genomics platform enables the discovery of these cell-type-specific selection dynamics from native human samples. Alternatively, recent advances in mitochondrial base editing may provide a scalable platform for evaluating dynamics of installed mutations (37). Moving forward, a combination of these approaches may enable characterizing the extent of potential functional synonymous variation throughout the mitochondrial genome and in diverse cellular contexts.

Though reports in model organisms have described nuclear-encoded tRNA transfer to the mitochondria to compensate for deficiencies of the mitochondrial tRNA pool, to the best of our knowledge, no evidence of functional nuclear tRNAs in human mitochondria has been observed. The pathogenicity of LOF mtDNA-encoded tRNA mutations in MELAS (m.3243 in a leucine tRNA), MERRF (m.8344 in lysine tRNA), and other mitochondriopathies further suggest a lack of meaningful nuclear tRNA contribution to mitochondria in humans (38). Thus, the translation of the 13 mtDNA-derived polypeptides utilizes only the 22 tRNAs encoded in the mitochondrial genome. Consistent with this concept, m.7076A > G requires wobble-dependent translation via mt-tRNA^Gly^ at the *MT-CO1*:p.Gly391 polypeptide, which occurs but leads to ribosome stalling and diminished CD8^+^ T cell fitness. Notably, this variant does not impact the abundance of the *MT-CO1* mRNA in any profiled cell state, providing an independent and distinct mechanism from a recent report of functional synonymous variation that impacts mRNA stability in yeast (39).

In bacteria, selection for codon:anticodon matching is strongest in species that are the fastest growing (40). Similarly, our inferences of human cell states are associated with rapid proliferation, specifically CD8^+^ SLECs, which are biased toward optimized codon syntax. Noting that mitochondria emerged from bacterial ancestors via endosymbiosis (41), our data convey a model where similar selective pressures in mitochondria may emerge in states of high metabolic demands, including CD8^+^ T cell differentiation/activation, or with even small competitive advantage contributing to tumorogenesis and tissue evolution. With the continued application of single-cell DNA sequencing with concomitant cell state characterization, we anticipate that our multiomics approach will continue to reveal unique insights into the contribution of DNA variation to human phenotypes in distinct cellular and evolutionary contexts. More generally, our approach motivates the continued study of heterogeneous tissues and donors with single-cell sequencing to realize patterns of somatic evolution that will complement our understanding of gain- and loss-of-function mutations beyond annotations in cancer genomes.

## Materials and Methods

### Donor Identification.

Donor 1 is a healthy male donor whose blood was drawn under Stanford Human Subjects Research IRB #14734. The full study protocol for single-cell sequencing was approved, and the donor was recruited with written consent. No apparent phenotype was discernible from this individual. After analysis of germline mtDNA, this individual is part of Haplogroup M (specifically, M38a) as determined by Haplogrep 3 (42) with a 96% confidence. This haplogroup includes m.7076A as part of its defining variants.

### Mitochondrial Single-Cell ATAC-seq (mtscATAC-seq).

MtscATAC-seq libraries were generated using the 10× Chromium Controller and the Chromium Single Cell ATAC Library & Gel Bead Kit (#1000175) according to the manufacturer’s instructions (CG000209-Rev G; CG000168-Rev B) as outlined below and previously described to increase mtDNA yield and genome coverage (7). Briefly, 1.5 ml or 2 ml DNA LoBind tubes (Eppendorf) were used to wash cells in PBS and downstream processing steps. After washing cells were fixed in 1% formaldehyde (FA; ThermoFisher #28906) in PBS for 10 min at RT, quenched with glycine solution to a final concentration of 0.125 M before washing cells twice in PBS via centrifugation at 400 g, 5 min, 4 °C. Cells were subsequently treated with lysis buffer (10 mM Tris-HCl pH 7.4, 10 mM NaCl, 3 mM MgCl_2_, 0.1% NP40, 1% BSA) for 3 min for primary cells on ice, followed by adding 1 ml of chilled wash buffer and inversion (10 mM Tris-HCl pH 7.4, 10 mM NaCl, 3 mM MgCl_2_, 1% BSA) before centrifugation at 500 g, 5 min, 4 °C. The supernatant was discarded and cells were diluted in 1× Diluted Nuclei buffer (10× Genomics) before counting using Trypan Blue and a Countess II FL Automated Cell Counter. If large cell clumps were observed a 40 µm Flowmi cell strainer was used before processing cells according to the Chromium Single Cell ATAC Solution user guide with no additional modifications. Briefly, after tagmentation, the cells were loaded on a Chromium controller Single-Cell Instrument to generate single-cell Gel Bead-In-Emulsions (GEMs) followed by linear PCR as described in the protocol using a C1000 Touch Thermal cycler with 96-Deep Well Reaction Module (BioRad). After breaking the GEMs, the barcoded tagmented DNA was purified and further amplified to enable sample indexing and enrichment of scATAC-seq libraries. The final libraries were quantified using a Qubit dsDNA HS Assay kit (Invitrogen) and a High Sensitivity DNA chip run on a Bioanalyzer 2100 system (Agilent).

### Single-Cell RNA-Seq and TCR Profiling.

Libraries for scRNA-seq were generated using the 10× Chromium Controller and the Chromium Single Cell 5′ Library Construction Kit and human B cell and T cell V(D)J enrichment kit according to the manufacturer’s instructions. Briefly, the suspended cells were loaded on a Chromium controller Single-Cell Instrument to generate single-cell Gel Bead-In-Emulsions (GEMs) followed by reverse transcription and sample indexing using a C1000 Touch Thermal cycler with 96-Deep Well Reaction Module (BioRad). After breaking the GEMs, the barcoded cDNA was purified and amplified, followed by fragmenting, A-tailing, and ligation with adaptors. Finally, PCR amplification was performed to enable sample indexing and enrichment of scRNA-seq libraries. For T cell receptor sequencing, target enrichment from cDNA was conducted according to the manufacturer’s instructions. The final libraries were quantified using a Qubit dsDNA HS Assay kit (Invitrogen) and a High Sensitivity DNA chip run on a Bioanalyzer 2100 system (Agilent). 10× scRNA-seq libraries were sequenced as recommended by the manufacturer (~20,000 reads per cell) via a NovaSeq 6000 using an S4 flow cell.

### ATAC With Selected Antigen Profiling By Sequencing (ASAP-seq).

Cultured primary T cells were stained with a TSA-conjugated antibody panel (BioLegend “Universal” Totalseq-A panel) that targets 154 distinct epitopes as previously described (9). Briefly, following sorting, cells were fixed in 1% formaldehyde and processed as described for the mtscATAC-seq workflow described above, with the modification that during the barcoding reaction, 0.5 µl of 1 µM bridge oligo A (BOA for TSA) was added to the barcoding mix. For GEM incubation the standard thermocycler conditions were used as described by 10× Genomics for scATAC-seq. Silane bead elution and SPRI cleanup steps were modified as described to generate the indexed protein tag library (9). The final libraries were quantified using a Qubit dsDNA HS Assay kit (Invitrogen) and a High Sensitivity DNA chip run on a Bioanalyzer 2100 system (Agilent). Libraries were amplified, sequenced, and preprocessed as previously described (9).

### Single-cell ATAC-Seq Analyses.

Raw sequencing data were demultiplexed using CellRanger-ATAC mkfastq. Demultiplexed sequencing reads for all libraries were aligned to the mtDNA blacklist modified (7) hg38 reference genome using CellRanger-ATAC count v2.0. Mitochondrial DNA genotypes were determined using the mgatk workflow with default parameters (7). Notably, as nuclear-encoded mitochondrial DNA (NUMTs) are generally not in accessible chromatin, our prior analyses indicate that NUMT contamination in this assay is less than 1 in 1,000 fragments, reflecting a minimal potential contamination for these analyses. Cell state analyses, including gene activity scores and surface protein visualization, were performed using the Seurat/Signac framework (43, 44). For PBMC cell type annotations, granular cell type labels and UMAP coordinates were established by using the Seurat Dictionary Learning (45) for cross-modality integration. We used Azimuth CITE-seq reference dataset labels (46) with public 10× Genomics Multiome RNA- and ATAC-seq PBMC data as a cross-modality bridge. To assess whether additional genetic heterogeneity was present in cells marked with m.7076A > G, we split the full single-cell .bam file based on high-confidence cells with either the m.7076A or m.7076G allele and used FreeBayes (47) to genotype variants in the nuclear chromosomes using pseudobulk .bam files of the scATAC-seq profiles. After intersecting the two .vcf files from either the m.7076A or m.7076G variant calls, we did not identify evidence of high-quality variants (QUAL>100) specific to either allele, noting that our variant calling was restricted to accessible chromatin regions.

### Single-Cell RNA-Seq Analyses.

Raw sequencing data were demultiplexed using CellRanger mkfastq and aligned to the host reference genome using CellRanger v6.0 and TCR sequences were processed using the CellRanger vdj pipeline with default settings. Mitochondrial DNA genotypes were determined using the mgatk workflow with default parameters (7) while accounting for UMIs using the -ub tag. T cell subset annotations were derived using the Azimuth CITE-seq reference dataset labels (46) from healthy 10× PBMC data. Cells were assigned as m.7076A or m.7076G using a minimum coverage of 2 UMIs and homoplasmy for either allele from the transcriptomic data (mean coverage = 3.2×/cell from 5’ scRNA-seq for m.7076). Expression levels of *MT-CO1* were determined from the default normalization in Seurat and assessed for differences using a Wilcoxon test with the Benjamini–Hochberg adjustment for multiple testing.

### SCENITH Assay.

The SCENITH assay was performed as previously described (28) with minor modifications. In brief, cryopreserved PBMCs were thawed in a 37 °C water bath prior to serial dilution with prewarmed media, i.e. human plasma-like media (HPLM, Gibco) supplemented with 10% fetal bovine serum (FBS, PanBiotech) and 1% penicillin/streptomycin (P/S, PanBiotech). The cells were washed twice with media and centrifuged at 400 g for 5 min at 4 °C. Subsequently, 2 × 10^5^ PBMCs per condition were incubated for 30 min with either 100 mM 2-Deoxy-D-Glucose (“DG”, Sigma), 1 μM Oligomycin A (“oligo”, Sigma), a combination of both inhibitors (“DGO”), or left untreated at 37 °C with 5% CO_2_. Subsequently, 10 μg/mL puromycin dihydrochloride (Gibco) and Brefeldin A (Gibco) were added to all conditions (except puromycin negative controls) for an additional incubation of 30 min under the same conditions. The cells were then washed twice (400 g, 4 °C, 5 min) in phosphate-buffered saline (PBS, Gibco) containing 0.1% bovine serum albumin (BSA, Miltenyi Biotec) and stained with the fixable fluorescent viability dye eFluor780 (Thermo Fisher Scientific) for 10 min on ice. After an additional wash, the cells were incubated with Monocyte and FcX Blocking Solutions (BioLegend) for 10 min on ice. Surface staining was performed with anti-human CD3-Alexa Fluor 488 (clone OKT3, BioLegend), anti-human CD8a-Brilliant Violet 711 (clone RPA-T8, BioLegend), anti-human CD45RA-BUV395 (clone 5H9, BD Biosciences), anti-human CD62L-PE (clone DREG-56, BioLegend), anti-human CD127-Brilliant Violet 510 (clone A019D5, BioLegend), anti-human CD197-Brilliant Violet 605 (clone G043H7, BioLegend), and anti-human KLRG1-PE Vio770 (clone REA261, Miltenyi Biotec) in PBS/0.1% BSA for 30 min at 4 °C. After washing (400 g, 4 °C, 5 min), the cells were fixed and permeabilized using the eBioscience Foxp3/Transcription Factor Staining Buffer Set (Thermo Fisher Scientific) according to the manufacturer’s instructions. Following fixation, the cells were washed twice (500 g, 4 °C, 5 min) with Permeabilization Buffer (Thermo Fisher Scientific) and incubated with Monocyte and FcX Blocking Solutions for 15 min at room temperature. Intracellular staining was then performed with anti-human puromycin-Alexa Fluor 647 (clone 12D10, Merck) for 30 min on ice. After a final washing step with Permeabilization Buffer (500 g, 4 °C, 5 min), the cells were resuspended in PBS and stored on ice until analysis using a BD LSR Fortessa X-20 flow cytometer.

### Mitochondrial Ribosome Profiling.

PBMCs were cultured in RPMI-1640 medium supplemented with 10% fetal bovine serum (FBS), penicillin and streptomycin, and 10 ng/ml IL-2 (PeproTech) at 37 °C, 5% CO_2_. For in vitro expansion, cells were stimulated with Dynabeads Human T-Activator αCD3/αCD28 at a bead-to-cell ratio of 1:2 (11131D, Thermo Fisher Scientific). Cell counts were determined every 2 to 3 d, and maintained at a density of 1 to 2 × 10^6^ cells/ml. On day 7 after stimulation, cells were collected for mitochondrial ribosome profiling (MitoRiboSeq). Briefly, 0.1 mg/ml cycloheximide (CHX) and 0.1 mg/ml chloramphenicol (CAP) were directly added into the cell culture media followed by 5 min incubation at 37 °C, 5% CO_2_. 20 × 10^6^ cells were then centrifuged and washed with ice-cold PBS supplied with 0.1 mg/ml CHX and 0.1 mg/ml CAP. Dynabeads were magnetically removed, cells were pelleted, snap-frozen in liquid nitrogen, and stored at −80 °C until further processing.

The protocol for mitochondrial ribosome enrichment was adapted from previous work (29). The flash-frozen cell pellet was resuspended in 1 mL of lysis buffer (20 mM Tris-HCl pH 8.0, 100 mM KCl, 10 mM MgCl2, 0.1 mg/mL chloramphenicol, 0.1 mg/mL cycloheximide, 1 mM DTT, 1% (vol/vol) Triton X-100, 0.1% (vol/vol) NP-40, 1× complete protease inhibitor cocktail, 20 U/mL RNasin, 20 U/mL DNase I) and incubated for 10 min on ice. The lysate was homogenized by passing it 3 times through a 26 G needle. Cell debris was removed by centrifugation at 16,000 g for 10 min (4 °C) and 100 µL of clarified lysate was kept aside for preparation of RNA-seq libraries. RPFs were generated by nuclease digestion. To this end, 3.75 U/µL of micrococcal nuclease (NEB) and 5 mM CaCl2 were added to the remaining cell lysate; the incubation was performed at room temperature with gentle shaking for 1 h. The reaction was quenched by adding 6 mM of EGTA. The digested lysate was loaded on top of a linear 5 to 45% sucrose gradient containing 20 mM Tris-HCl pH 8.0, 100 mM KCl, 10 mM MgCl2, 0.1 mg/mL chloramphenicol, 0.1 mg/mL cycloheximide, and 1 mM DTT and centrifuged at 35,000 rpm, 4 °C for 2 h using a Beckmann SW40 rotor. The gradient was then fractionated into 20 fractions of 0.57 mL using a Biocomp gradient station fractionator, which allowed the recording of a UV absorbance profile at 254 nm.

### Western Blot of Gradient Fractions.

Western blot analysis was used to monitor the successful isolation of the 55S ribosome. Proteins were precipitated from 230 µL of each gradient fraction and 25 µL of input lysate by adding Trichloroacetic acid (TCA) to a final concentration of 20%. Samples were incubated on ice for 1 to 2 h and proteins were precipitated by centrifugation at 15,000 g at 4 °C. The supernatant was removed and protein pellets were washed twice with 300 µL ice-cold acetone, followed by a 15 min centrifuging at 15,000 g and 4 °C. Pellets were air-dried and resuspended in 30 fL 1× Lämmli-Buffer. Samples were separated on a Tris SDS-PAGE gel and transferred to a nitrocellulose membrane using the iBlot/iBind system (Thermo Fisher Scientific). The presence of mitochondrial and cytoplasmic ribosomes in gradient fractions was estimated by incubating resulting membranes with anti-MRPL11 monoclonal antibody (D68F2, Cell Signaling) and anti-RPS6 rabbit monoclonal antibody (Cell Signaling).

### RPF Isolation and Library Generation.

RPFs were isolated using Phenol/Chloroform extraction. To that end, 300 µl of the corresponding gradient fractions were mixed with equal amounts of Phenol:Chloroform:IAA (25:24:1, pH 6.6), transferred to a PhaseLock tube (VWR International), and centrifuged at 15,000 g for 5 min. A second clean-up step was performed using the isolated aqueous phase and adding equal amounts of Chloroform. After centrifugation, the aqueous phase was isolated and purified using the RNA Clean & Concentrator kit (Zymo Research) by following the manufacturer’s instructions for small RNAs. Subsequent library preparation was performed as previously described (48) with the following modification: Ribosomal RNA depletion was skipped for RPF libraries due to low starting material. For the preparation of matched RNA-seq libraries, total RNA was extracted from 40 µL of clarified input lysate using the Direct-zol RNA MiniPrep Kit (Zymo Research). Subsequently, rRNA was depleted using riboPOOLs (siTOOLs Biotech) and following the manufacturer’s instructions. Subsequently, RNA-seq libraries were generated as described previously (48).

Raw .fastq files were trimmed for adapter sequences using cutadapt and subsequently aligned with bwa mem using default parameters as previously described (48). Summary statistics of coverage and m.7076A > G heteroplasmy were determined using the mgatk (7) workflow with default parameters as well as the -kd flag to retain duplicate fragments with the same start and end coordinates. Pileup coverages of the locus split by the m.7076 allele were determined using a custom python script using pysam library. Statistical comparisons of libraries were performed using Fisher’s exact tests.

### Mitochondrial and Nuclear Exome Sequencing.

Cryopreserved PBMCs were thawed in a 37 °C water bath before serial dilution with prewarmed RPMI-1640 Medium (Gibco) containing 10% FBS and 1% P/S (PanBiotech). The cells were centrifuged at 400 g for 5 min at 4 °C and resuspended in ice-cold PBS (Gibco) with 0.5% BSA (Miltenyi Biotec) and 2 mM EDTA (Invitrogen). Cell numbers and viability were determined with an Invitrogen Countess 3 before cells were centrifuged again (400 g, 4 °C, 5 min). Subsequently, Fc-receptors were blocked with FcX Blocking Solution (BioLegend) for 5 min on ice. Surface marker staining was then performed for 30 min on ice with the following antibodies: anti-human CD3-Alexa Fluor 488 (clone OKT3, BioLegend), anti-human CD4-PE (clone RPA-T4, BioLegend), anti-human CD8a-Brilliant Violet 711 (clone RPA-T8, BioLegend), anti-human CD14-PerCP/Cyanine5.5 (clone 63D3, BioLegend), anti-human CD25-Alexa Fluor 647 (clone M-A251, BioLegend), anti-human CD45RA-Brilliant Violet 785 (clone HI100, BioLegend), anti-human CD45RO-APC/Cyanine7 (clone UCHL1, BioLegend), anti-human CD127-Brilliant Violet 510 (clone A019D5, BioLegend), anti-human CD197-Brilliant Violet 605 (clone G043H7, BioLegend), and anti-human KLRG1-PE Vio770 (clone REA261, Miltenyi Biotec). Stained PBMCs were washed twice with PBS/0.5%BSA/2 mM EDTA and stained with the viability dye SYTOX Blue (Invitrogen). Subsequently, CD3^+^CD8^+^CD45RA^+^KLRG1^+^CD127^low^ short-lived effector cells (SLECs), CD3^+^CD8^+^CD45RO^+^KLRG1^+^ CD127^low^ TEM cells, CD3^+^CD4^+^CD45RO^+^ T memory cells, and CD14^+^ monocytes were sorted using a BD FACSAria Fusion.

The DNA from sorted cell populations was extracted with a DNeasy Blood & Tissue Kit (Qiagen) according to the manufacturer’s instructions. Whole exome sequencing libraries were prepared using the Agilent SureSelect XT HS2 library preparation kit with Human All Exon V8 + Mito (500:1 ratio) enrichment baits, starting from 38 to 68 ng of input DNA, according to the manufacturer’s protocol. Final libraries were pooled at equimolar concentrations and sequenced on a single lane of an Illumina NovaSeq X Plus 10B flow cell using the paired-end 100 base pair (PE100) mode.

Exome variants were called using the .fastq to .vcf framework available in GATK Best Practices (49). In brief, adapters were trimmed and reads were aligned using bwa (50). PCR duplicates were removed with Picard and variant quality recalibration and calling was performed with GATK. Population allele frequencies were estimated using bam-readcount per library (51). Using the strategy in *SI Appendix*, Fig. S3 *D* and *E*, we attempted to identify nuclear variants associated with either gain- or loss- of function in the m.7076A or G populations using thresholds to reflect the putative fraction from each bulk-sorted population. Though challenging to verify a negative result, our inclusive filters suggest that no nuclear variants in the exome can readily explain the selection phenotype.

### Annotation of Synonymous Variation in the Mitochondrial Genome.

The revised Cambridge reference sequence (used in GRCh37, GRCh38, and hg38) was used as the basis for the ontogeny of somatic variation. We used all possible mtDNA variations and protein-coding annotations as previously described (8). Using this landscape of 8,284 synonymous mutations, we annotated whether the codon in the reference or the alternate allele would create a canonical (i.e., Watson–Crick–Franklin, WCF) base-pairing between the codon and anticodon or would require wobble-dependent base-pairing for translation. Once these annotations were established, the null model of the abundance of each variant class (*SI Appendix*, Fig. S5) was derived from the empirical distribution of these variant classes in the full mitochondrial genome. Notably, for the 12 protein-coding genes on the H strand, the proportion of codons requiring the wobble effect for translation was markedly lower (range: 47.3 to 57%) than *MT-ND6* (only L strand gene; 74.9%), reflecting the intrinsic sequence bias of the mtDNA genome.

DNA variants from the mitochondrial genome used to define haplogroups were determined from the PhyloTree Build 17 annotation as distributed by HaploGrep2 (52, 53). Filtered variants required a phylogenetic recurrence of 10, leading to 289 variants, including 48 missense and 87 synonymous variants. To calculate the interspecies evolution of the mitochondrial genome, we used the phyloP annotation per nucleotide (rather than per variant and this metric is not available from phyloP), requiring our annotation of wobble-position nucleotides in the mitochondrial genome to be assigned to one of three categories (*SI Appendix*, Fig. S5*E*). The associations shown in *SI Appendix*, Fig. S5*E* are for a 20-species phyloP calculation but were consistent using a 100-species estimation as well. Both phyloP annotations were downloaded from UCSC Genome Browser for the GRCh38 genome annotation. Similar analyses for the murine mm10 genome, including a 100-species phyloP, are shown in *SI Appendix*, Fig. S5.

## Supplementary Material

Appendix 01 (PDF)

## Data Availability

Sequencing data associated with this work is available at GEO accession GSE216915 (54). Custom code to reproduce all analyses supporting this manuscript is available at https://github.com/caleblareau/7076 (55). All study data are included in the article and/or *SI Appendix*.
