# Supplementary material for: Cell type–specific purifying selection of synonymous mitochondrial DNA variation
Source: Proc Natl Acad Sci U S A. 2025 Jul 24;122(30):e2505704122. doi: 10.1073/pnas.2505704122 (PMC12318227; doi:10.1073/pnas.2505704122)
Supplement: Supplementary file 1 — Appendix 01 (PDF) [file pnas.2505704122.sapp.pdf]

Supplemental Information for:

Cell type-specific purifying selection of synonymous mitochondrial DNA variation

Caleb A. Lareau[1,2,*,+], Patrick Maschmeyer[3,4,*], Yajie Yin[2], Jacob C. Gutierrez[1,2], Ryan S. Dhindsa[5,6,7], Anne-Sophie Gribling-Burrer[8], Sebastian Zielinski[8], Yu-Hsin Hsieh[3,4], Lena Nitsch[3,4,9], Veronika Dimitrova[3,4], Benan Nalbant[1], Frank A. Buquicchio[2], Tsion Abay[2,10], Robert R. Stickels[2], Jacob C. Ulirsch[11], Patrick Yan[2], Fangyi Wang[2], Zhuang Miao[2,12], Katalin Sandor[2], Bence Daniel[2], Vincent Liu[2,12], Paul L. Mendez[9], Petra Knaus[9], Manpreet Meyer[3,4,13,14], William J. Greenleaf[12], Anshul Kundaje[12,15], Redmond P. Smyth[8,16], Mathias Munschauer[8,16,17], Leif S. Ludwig[3,4,+], Ansuman T. Satpathy[2,18,+]

1. Computational and Systems Biology Program, Memorial Sloan Kettering Cancer Center, New York, NY, USA
2. Department of Pathology, Stanford University, Stanford CA, USA
3. Berlin Institute of Health at Charité – Universitätsmedizin Berlin, Berlin, Germany
4. Max-Delbrück-Center for Molecular Medicine in the Helmholtz Association (MDC), Berlin Institute for Medical Systems Biology (BIMSB), Berlin, Germany
5. Department of Pathology and Immunology, Baylor College of Medicine, Houston, TX, USA
6. Jan and Dan Duncan Neurological Research Institute, Texas Children's Hospital, Houston, TX, USA
7. Department of Molecular and Human Genetics, Baylor College of Medicine, Houston, TX, USA
8. Helmholtz Institute for RNA-based Infection Research, Helmholtz-Center for Infection Research, Würzburg, Germany
9. Department of Biology, Chemistry, Pharmacy, Freie Universität Berlin, Berlin, Germany
10. Biological and Biomedical Sciences Program, Harvard Medical School, Boston, MA, USA
11. Illumina Artificial Intelligence Laboratory, Illumina, San Diego, CA, USA
12. Department of Genetics, Stanford University, Stanford CA, USA
13. Department of Biology, Chemistry, Pharmacy, Freie Universität Berlin, Berlin, Germany
14. Boston Children's Hospital and Harvard Medical School, Boston, MA, USA
15. Department of Computer Science, Stanford University, Stanford, CA, USA
16. Department of Infectious Diseases, Center for Integrative Infectious Disease Research, Heidelberg, Germany
17. Department of Medicine, Heidelberg University, Heidelberg, Germany
18. Parker Institute for Cancer Immunotherapy, San Francisco, CA, USA

* Equal contributions

\+ Correspondence to: lareauc@mskcc.org; leif.ludwig@bih-charite.de; satpathy@stanford.edu

This PDF File contains Figures S1-S5

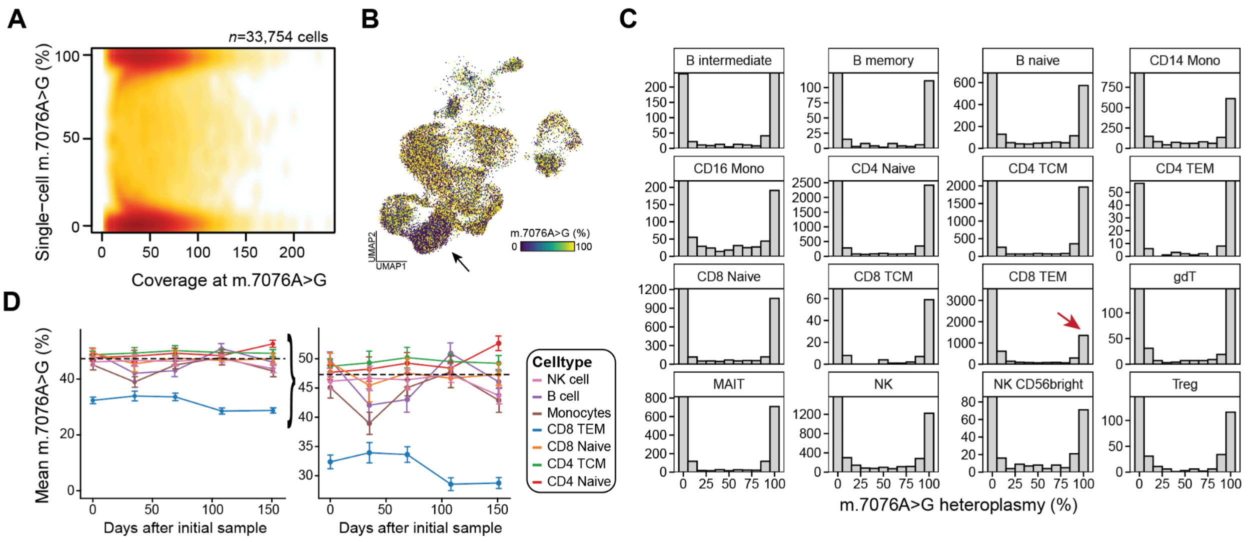

Fig. S1. Supporting information for selection against mutant m.7076G in CD8⁺ TEM cells. (A) Heatmap of single-cells comparing the coverage of both m.7076 alleles against heteroplasmy. **(B)** Compare to **Fig. 1E** with the smoothed representation. **(C)** Histograms comparing the 16 most common cell types from the Azimuth/Bridge Integration annotation. The red arrow highlights the significant reduction of cells with the m.7076A>G variant specifically in CD8⁺ TEM cells, but not other cell types. **(D)** Longitudinal heteroplasmy of different cell populations over >150 days of sampling. The right panel is a zoom of the region on the left panel (note axis). Pseudobulk heteroplasmy estimates including the standard error of the mean are shown.

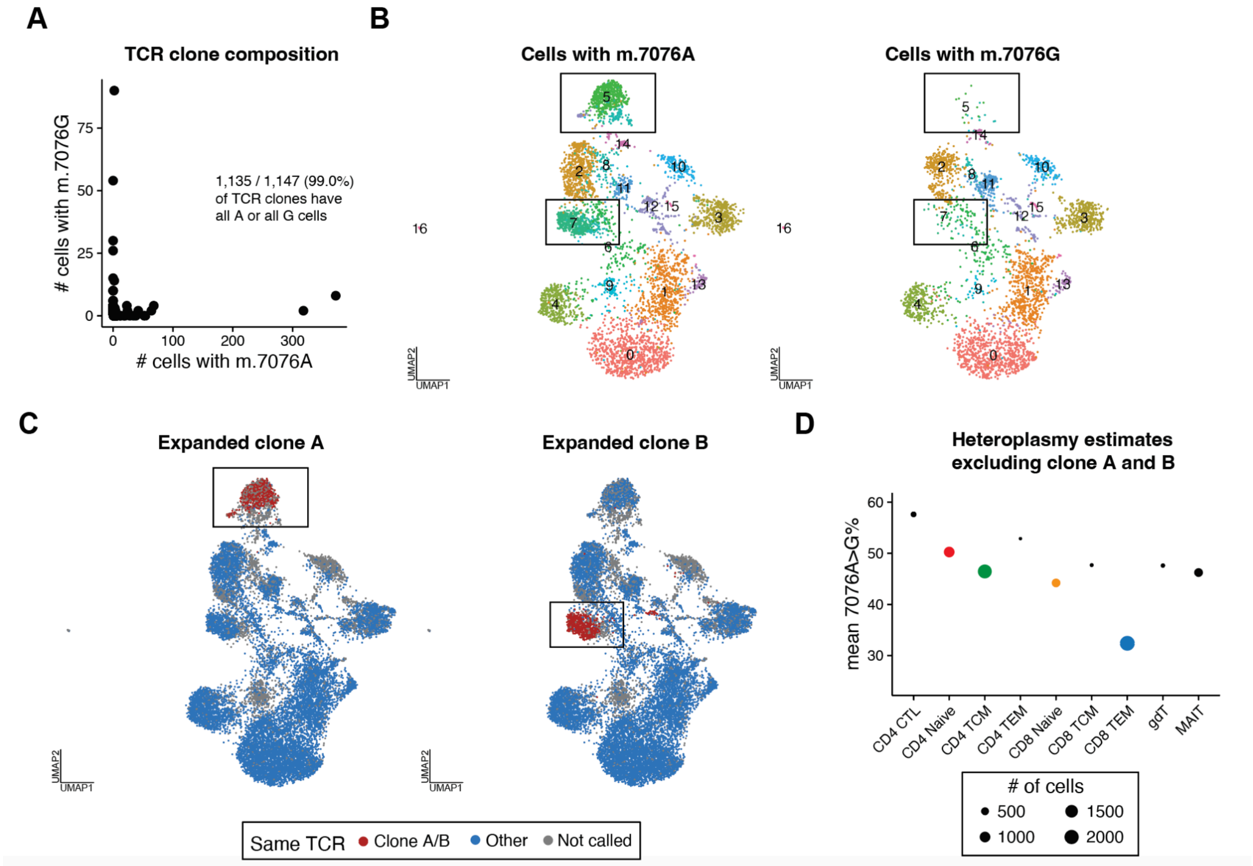

Fig. S2. Supporting information for selection against mutant m.7076A>G using scRNA-seq. (A) Overlap of TCR clones with respective m.7076 alleles. Each dot is a TCR clone (min. 2 cells) summarizing the number of cells with either wildtype m.7076A or mutant m.7076G homoplasmy. **(B)** Unsupervised clustering and dimensionality reduction of T cells stratified by m.7076A or m.7076G homoplasmy. Black boxes around clusters 5 (98.1%) and 7 (95.9%) represent specific cell states that are primarily restricted to cells harboring the m.7076A allele. **(C)** Annotation of two highly expanded TCR clones restricted to cells homoplasmic for wildtype m.7076A. Blue represents TCR clones (n≥2 cells) that were not highly expanded. **(D)** Heteroplasmy of the m.7076A>G allele in indicated T cell subpopulations based on scRNA-seq after excluding clones A and B from panel (C). The size of each dot is scaled by the abundance of cells in each cell state.

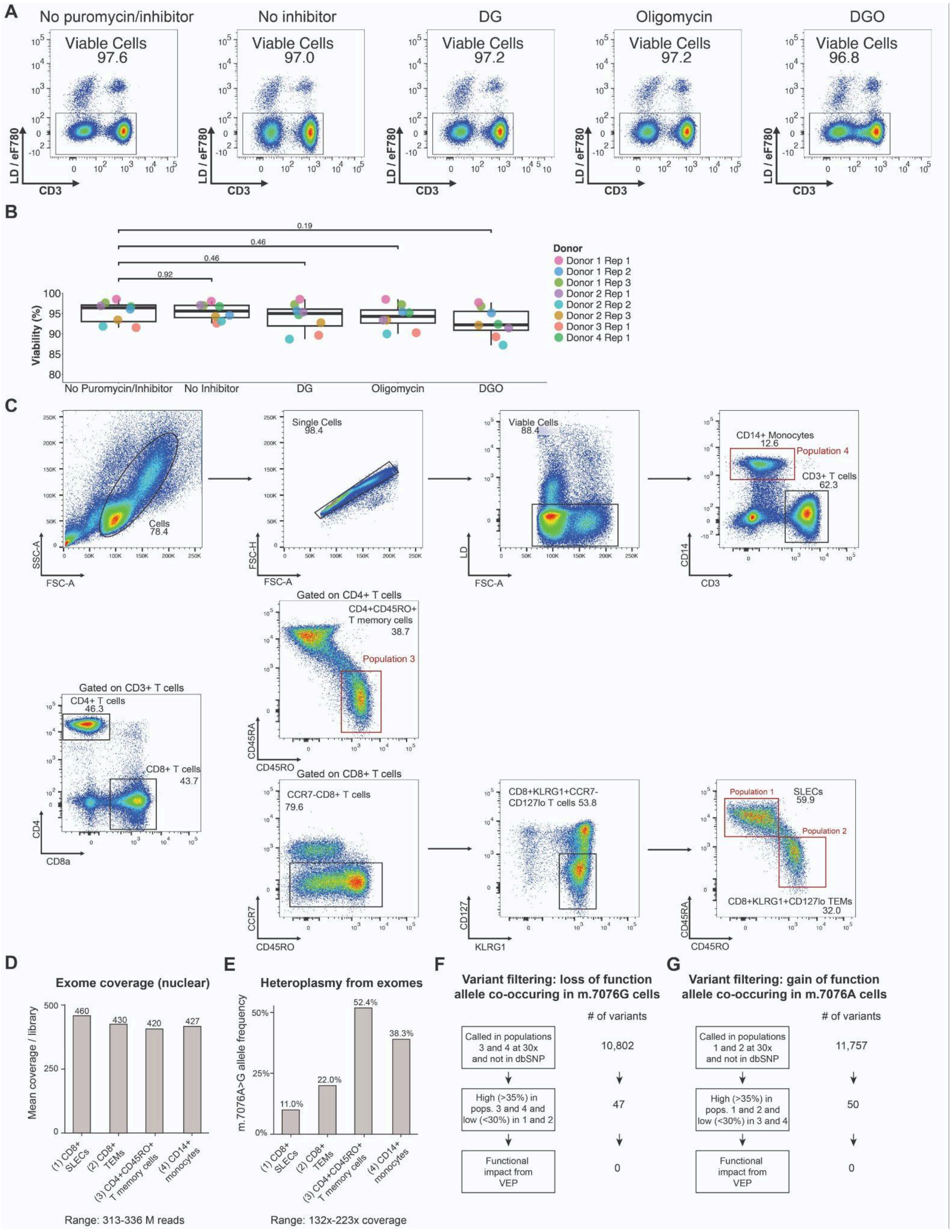

Fig. S3. Supporting information SCENITH and characterization of potential nuclear variants co-segregating with m.7076A>G. (A) Representative FACS plots showing % viable (i.e., LD/eF780-negative) lymphocytes, across different SCENITH treatment conditions: no treatment (“No puromycin/inhibitor”), puromycin alone (“No inhibitor”), or puromycin combined

with metabolic inhibitors targeting glycolysis (“DG”), OXPHOS (“Oligomycin”), or both pathways (“DGO”). All displayed cells are gated on lymphocyte singlets. **(B)** Boxplots summarizing lymphocyte viability for all indicated donors and technical replicates. P-values were calculated using a two-sided Wilcoxon test. **(C)** Summary of flow cytometry isolation of specific populations expected to be enriched (populations 3,4) or depleted (populations 1,2) of m.7076A>G for downstream whole-exome sequencing analysis. **(D)** Summary of exome coverage across the nuclear genome for four libraries. **(E)** m.7076A>G heteroplasmy from exome libraries of sorted populations. **(F)** Logic for identifying candidate mutations co-occurring with m.7076G. **(G)** Logic for identifying candidate mutations co-occurring with m.7076A. No variants with predicted functional impact were identified.

A

Nuclear DNA codon use / bias table

Codon	Amino acid	Codon	Amino acid	Codon	Amino acid	Codon	Amino acid
TTT 0.99	Phe	TCT 1.18	Ser	TAT 0.95	Tyr	TGT 0.98	Cys
TTC 1.01		TCC 1.20		TAC 1.05		TGC 1.02	
TTA 0.54	Leu	TCA 0.99		TAA 0.83	Stop	TGA 1.51	Stop
TTG 0.83		TCG 0.28		TAG 0.66		TGG 1.00	Trp
CTT 0.87		CCT 1.22	Pro	CAT 0.90	His	CGT 0.49	Arg
CTC 1.09		CCC 1.20		CAC 1.10		CGC 0.94	
CTA 0.46		CCA 1.19		CAA 0.57	Gln	CGA 0.69	
CTG 2.21		CCG 0.39		CAG 1.43		CGG 1.14	
ATT 0.94	Ile	ACT 1.05	Thr	AAT 1.01	Asn	AGT 0.98	Ser
ATC 1.06		ACC 1.31		AAC 0.99		AGC 1.37	
ATA 1.00		ACA 1.22		AAA 0.93	Lys	AGA 1.44	Arg
ATG 1.00	Met	ACG 0.41		AAG 1.07		AGG 1.31	
GTT 0.80	Val	GCT 1.12	Ala	GAT 1.00	Asp	GGT 0.69	Gly
GTC 0.92		GCC 1.52		GAC 1.00		GGC 1.25	
GTA 0.52		GCA 1.01		GAA 0.92	Glu	GGA 1.09	
GTG 1.76		GCG 0.35		GAG 1.08		GGG 0.97	

B

Mitochondria DNA codon use / bias table

Codon	Amino acid	Codon	Amino acid	Codon	Amino acid	Codon	Amino acid
TTT 0.72	Phe (AAG)	TCT 0.69	Ser (AGU)	TAT 0.68	Tyr (AUG)	TGT 0.45	Cys (ACG)
TTC 1.28		TCC 2.19		TAC 1.32		TGC 1.55	
TTA 0.68	Leu (AAU)	TCA 1.82		TAA 1.71	Stop	TGA 1.77	Trp (ACU)
TTG 0.17		TCG 0.15		TAG 1.14		TGG 0.23	
CTT 0.61	Leu (GAU)	CCT 0.75	Pro (GGU)	CAT 0.37	His (GUG)	CGT 0.44	Arg (GCU)
CTC 1.56		CCC 2.17		CAC 1.63		CGC 1.65	
CTA 2.57		CCA 0.95		CAA 1.82	Gln (GUU)	CGA 1.78	
CTG 0.42		CCG 0.13		CAG 0.18		CGG 0.13	
ATT 0.78	Ile (UAG)	ACT 0.59	Thr (UGU)	AAT 0.39	Asn (UUG)	AGT 0.3	Ser (UCG)
ATC 1.23		ACC 1.77		AAC 1.61		AGC 0.84	
ATA 1.61	Met (UAC)	ACA 1.53		AAA 1.79	Lys (UUU)	AGA 0.57	Stop
ATG 0.39		ACG 0.11		AAG 0.21		AGG 0.57	
GTT 0.74	Val (CAU)	GCT 0.67	Ala (CGU)	GAT 0.45	Asp (CUG)	GGT 0.45	Gly (CCU)
GTC 1.15		GCC 1.95		GAC 1.55		GGC 1.64	
GTA 1.68		GCA 1.25		GAA 1.45	Glu (CUU)	GGA 1.26	
GTG 0.43		GCG 0.13		GAG 0.55		GGG 0.64	

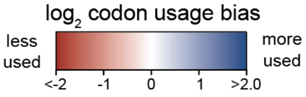

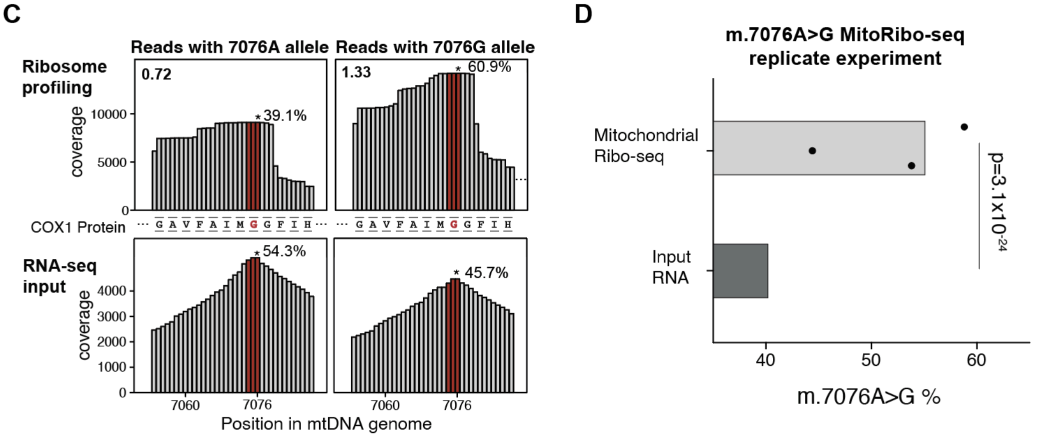

Fig. S4. Supporting information for analysis of the mitochondrial tRNA pool and impact on translational efficiency via the wobble effect. (A) Codon bias table for the nuclear genome. Within each amino acid, the ratio of observed codon usage over a null model of equal codon use per amino acid and colored by the $\log_2$ of this measure is shown. **(B)** Same as (A) but for the 13 polypeptides encoded in the mitochondrial genome. **(C)** Coverage near the m.7076A>G variant. Red bars indicate the mutated codon with the m.7076 allele (noted with an asterisk). The relative proportion of reads phased to either allele per library is indicated. The translation pause ratio, defined as the fraction of reads from ribosome profiling over the RNA-seq libraries, is noted in the top left corner. **(D)** Three additional replicate fractions of MitoRibo-seq from an independent experiment of Donor 1 cells. Statistical significance was determined using a Fisher's exact test of m.7076A and G alleles summed between replicates.

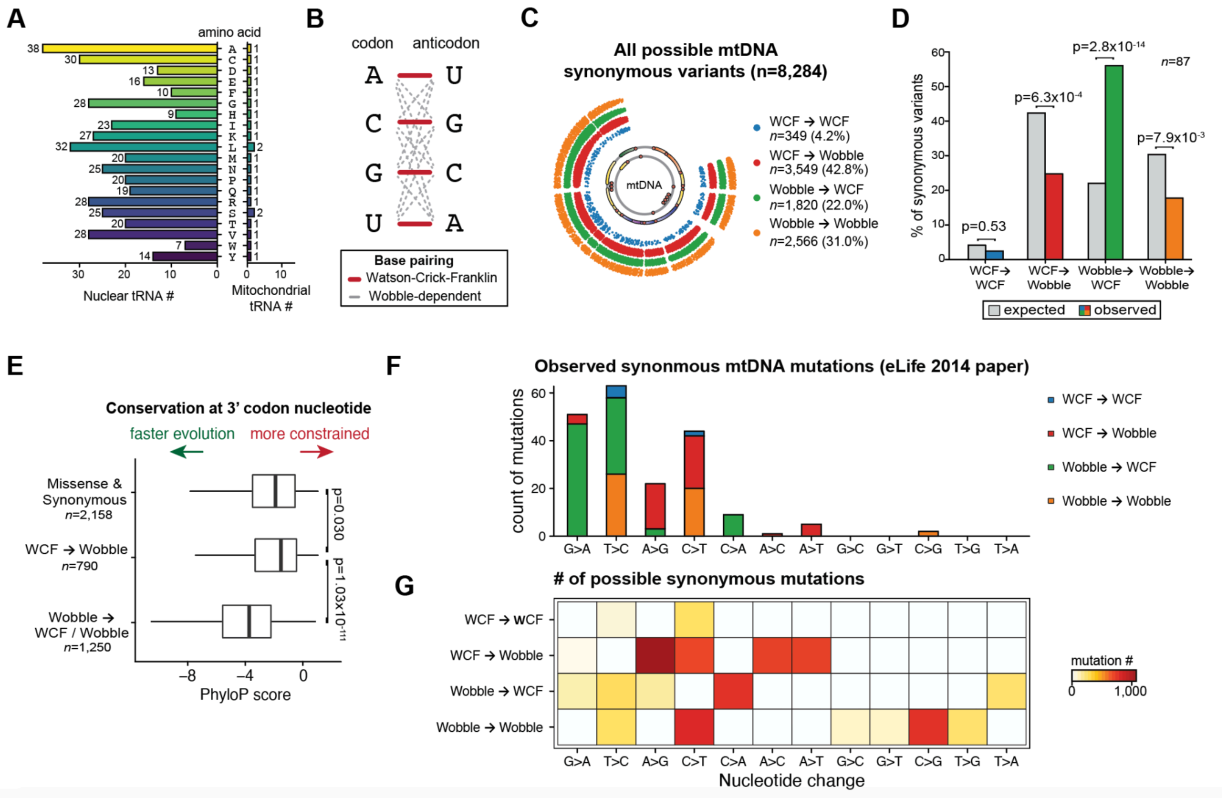

Figure S5. Systematic analysis of synonymous variation in the mtDNA genome. (A) Quantification of genes encoding tRNAs in the nuclear and mitochondrial genome. **(B)** Annotation of wobble-dependent base-pairing across all combinations of codon:anticodon interactions. **(C)** Classification of all 8,284 possible synonymous mtDNA variants based on Watson-Crick-Franklin (WCF) or wobble-dependent base-pairing at either the reference or alternative allele. **(D)** Comparison of % observed (color) versus expected (grey) for 87 haplogroup-defining, synonymous mtDNA mutations. P-values represent the statistical significance of a two-sided binomial test statistic. **(E)** Inter-species conservation at wobble-position nucleotides in mitochondrial codons. Reference alleles that can be mutated to different outcomes are specified and grouped with the number of wobble-position codons in each class noted. P-values represent a Wilcoxon test. **(F)** Count of somatic mtDNA mutations with synonymous mutation annotations from (32)**. (G)** Count of all possible synonymous mutations split by nucleotide change. Column arrangements match panel (F).
